# Cellular Localization and Characterization of Cytosolic Binding Partners for Gla Domain-containing Proteins PRRG4 and PRRG2*[Fn FN2]

**DOI:** 10.1074/jbc.M113.484683

**Published:** 2013-07-19

**Authors:** Mustafa N. Yazicioglu, Luca Monaldini, Kirk Chu, Fayaz R. Khazi, Samuel L. Murphy, Heshu Huang, Paris Margaritis, Katherine A. High

**Affiliations:** From the ‡ Division of Hematology, The Children's Hospital of Philadelphia, Philadelphia, Pennsylvania 19104,; the § Department of Pediatrics, Perelman School of Medicine at the University of Pennsylvania, Philadelphia, Pennsylvania 19104, and; the ¶ Howard Hughes Medical Institute, Chevy Chase, Maryland 20815

**Keywords:** Cell Signaling, Membrane Proteins, Molecular Biology, Mutagenesis Site-specific, Protein Carboxylation, Vitamin K, Cytosolic Binding Partners, Gla Domain, PRRG2, PRRG4

## Abstract

The genes encoding a family of proteins termed proline-rich γ-carboxyglutamic acid (PRRG) proteins were identified and characterized more than a decade ago, but their functions remain unknown. These novel membrane proteins have an extracellular γ-carboxyglutamic acid (Gla) protein domain and cytosolic WW binding motifs. We screened WW domain arrays for cytosolic binding partners for PRRG4 and identified novel protein-protein interactions for the protein. We also uncovered a new WW binding motif in PRRG4 that is essential for these newly found protein-protein interactions. Several of the PRRG-interacting proteins we identified are essential for a variety of physiologic processes. Our findings indicate possible novel and previously unidentified functions for PRRG proteins.

## Teaser

**Background:** PRRG proteins were cloned more than a decade ago, but their function is still not known.

**Results:** Several novel protein-protein interactions for PRRG are identified by array screening and pulldown analysis.

**Conclusion:** PRRG-initiated signaling events most likely depend on proteins with WW domains.

**Significance:** The protein-protein interactions identified here may help to elucidate the roles of PRRG proteins in different physiological settings.

## Introduction

The genes encoding the proline-rich γ-carboxyglutamic acid (PRRG)^2^ proteins were identified and characterized more than a decade ago. This family of γ-carboxyglutamic (Gla) domain-containing proteins has four members, and each has a hydrophobic domain, suggesting that they are membrane-bound (1, 2).

Gla domains were initially identified in blood coagulation proteins such as prothrombin, factor VII, factor IX, factor X, protein C, protein S, and protein Z. However, several proteins that are not involved in blood coagulation, including matrix Gla protein, osteocalcin, and Gas6, also contain Gla domains (3, 4). Although the expression of Gla proteins of the blood coagulation cascade is largely restricted to hepatic tissue, PRRG proteins are expressed in several extrahepatic sites (1, 2).

PRRG proteins are predicted to be ∼200 amino acids in length. They have Gla residues at the extracellular site, and possible SH3 and WW binding motifs in the cytosolic domains (2). The WW domain is among the smallest protein-protein interaction motifs. It consists of ∼40 amino acids with two highly conserved tryptophan residues that are spaced 20–23 amino acids apart (5, 6). Interactions mediated through WW domains have been associated with several diseases including cancer and neurological disorders (7–11).

Recent studies indicate a possible role for PRRG4 in disease. A study on WAGR (WWilms tumor, Aniridia, genitourinary malformations, and mental retardation) syndrome revealed that *PRRG4* is one of the genes deleted in all of the patients tested (12). Furthermore, a recent study on mesenteric lymph node (MLN) gene expression profiles of BALB/c mice in response to three common food allergens identified *PRRG4* as one of the top 10 up-regulated genes (13). A recent genome-wide association study to identify genes influencing Parkinson disease susceptibility showed that the SNP with the lowest *p* value lies between *PRRG4* and another gene (14).

There are only a handful of studies focused on *PRRG* genes and almost no studies addressing the biochemical nature of the protein itself. The only proteins that are known to interact with PRRG proteins are YAP1 and NEDD4, both of which can interact with PRRG2 (15, 16). NEDD4 is a ubiquitin ligase, and YAP1 is a downstream target of the Hippo tumor suppressor pathway (17).

To date, no data exist on the possible function and mechanism of PRRG proteins in human disease. We discovered by array screening and confirmed by GST pulldown and co-immunoprecipitation a number of novel protein-protein interactions for PRRG proteins. The protein-protein interactions identified here may help to elucidate PRRG functions in different physiological settings. We also used immunohistochemistry and subcellular fractionation to demonstrate localization of PRRG4 to the plasma membrane. Finally, we carried out quantitative analysis of PRRG2 and PRRG4 expression in different tissues; this may be useful for studies of the role of PRRG proteins. These findings contribute to an understanding of the role of PRRG proteins in disease pathology and the elucidation of their functions in different tissues.

## EXPERIMENTAL PROCEDURES

### 

#### 

##### Plasmids

The following plasmids were purchased from Addgene: FLAG-tagged YAP1, YAP2, YAP1 WW mutant, YAP1 S127A, YAP2 WW1 mutant, YAP2 WW2 (18), MAGI-1 (19), cool1/βPIX (20), 3×FLAG-tagged TAZ (∂393), TAZ WT, TAZ (∂WW), TAZ (S89A) (21). pCMV6-Entry (C-terminal Myc- and DDK-tagged) vector, Myc-DDK-tagged ORF clone of PRRG4, Lyn and FLAG-Myc-tagged OSTF, FLAG-Myc-tagged human PRRG4, WWTR1/TAZ, and GRB2 were purchased from OriGene. NEDD4L was purchased from Thermo Scientific. The plasmid catalogue numbers can be found in the supplemental Materials and Methods.

The cytosolic domain of PRRG4 from amino acids 138 to 226 was subcloned into PENTR/SD/D-TOPO vector (Invitrogen catalog number K2420-20) to make an entry vector. The entry vector was used to produce the N-terminal GST-tagged cytosolic domain of PRRG4 for bacterial expression vector Gateway pDEST15 (catalog number 11802-014) or mammalian expression vector Gateway pDEST 27 (catalog number 11812-013). Full-length PRRG4 was subcloned into a pcDNA3.1/V5-His (Invitrogen) vector for the cellular localization studies using immunohistochemistry.

##### GST Pulldown

BL21-AI One Shot chemically competent *Escherichia coli* (Invitrogen: catalog number C6070-03) was used for bacterial expression of the proteins. l-(+)-Arabinose (Sigma: catalog number A3256) was used to induce protein expression. GST-Bind^TM^ buffer kit (catalog number 70534-3) was purchased from EMD Biosciences and used according to manufacturer's protocol. Details of the experiment can be found in the supplemental Materials and Methods.

##### RNA Purification and Quantitative Real Time-PCR

RNA from tissues were purchased from Ambion. TaqMan® one-step RT-PCR master mix (catalog number 4309169) was used for quantitative real time PCR. TaqMan gene expression assays (catalog number 4331182) were used. Assay IDs are Hs00225378_m1 for human PRRG4, Hs00168745_m1 for human PRRG2, and Hs00172187_m1 for human POLR2A. The relative PRRG2 or PRRG4 expressions were acquired upon normalization of the results against POLR2A.

##### QuikChange Mutagenesis

We used QuikChange II XL site-directed mutagenesis kit (Stratagene catalog number 200522) to make the point mutations. The samples were prepared as 5 μl of 10× reaction buffer, 1 μl (50 ng) of dsDNA template, 1 μl (250 ng) of oligonucleotide primer #1 sense, 1 μl (250 ng) of oligonucleotide primer #2 antisense, 1 μl of dNTP mix, and double distilled H_2_O to a final volume of 50 μl, and 1 μl of *Pfu*Turbo DNA polymerase (2.5 units/μl). Cycling parameters for the QuikChange site-directed mutagenesis method were as follows: Step 1, 95 °C, 2 min; Step 2, 17 times (95 °C, 30 s; 55 °C, 1 min; 70 °C, 9 min 40 s); Step 3, 70 °C, 10 min; Step 4, 4 °C indefinitely. After PCR reaction, 1 μl of the DpnI restriction enzyme (10 units/μl) was added directly to each amplification reaction, and the samples were incubated at 37 °C for 1 h. 1 μl of the DpnI-treated samples was used to transform XL1-Blue supercompetent cells. Several colonies were picked and grown overnight in ampicillin-containing LB solutions. The samples were sequenced, and the clones that contained the desired mutation were selected. The sense sequence of the primer for Y189A mutation is: 5′-GGA GGA TGC AGG ATT ACC TTC TGC TGA ACA GGC AGT GGC GC-3′, and the antisense sequence of the primer for Y189A mutation is: 5′-GCG CCA CTG CCT GTT CAG CAG AAG GTA ATC CTG CAT CCT CC-3′. The sense sequence of the primer for Y207A is: 5′-CAG TGT TTC ACC ACC ACC ACC AGC TCC TGG GCA CAC-3′, and the antisense sequence of the primer for Y207A mutation is: 5′-GTG TGC CCA GGA GCT GGT GGT GGT GGT GAA ACA CTG-3′.

##### Cell Lines

HEK293 cell line was a gift from Research Vector Core Facility at The Children's Hospital of Philadelphia. The cells were grown at 95% air and 5% carbon dioxide (CO_2_) conditions at 37 °C. Fetal bovine serum to a final concentration of 10% was added to DMEM (Invitrogen, catalog number 11965).

For the experiments involving detection of post-translational modification of PRRG4 Gla domain upon vitamin K treatment, HEK293 cells were initially transfected with full-length PRRG4. The following day, the medium was changed to no FBS-containing DMEM supplemented with 1× insulin/transferrin/sodium selenite with the addition of vitamin K (20 μg/ml) or warfarin (300 μg/ml). After 2 days of incubation, the samples were lysed and pulled down with V5 or Gla antibodies. Immunoblotting was done with anti-V5 antibodies.

##### Immunoblotting

Samples were prepared with NuPAGE® LDS sample buffer (catalog number NP0007) and NuPAGE reducing agents (catalog number NP0004). SeeBlue Plus2 (Invitrogen catalog number LC5925) was used as protein marker. Samples were run on the NuPAGE Novex 12% or 4–12% Bis-Tris gels (catalog numbers NP0321BOX, NP0323BOX, NP0323BOX, NP0341BOX, NP0342BOX, and NP0343BOX) and transferred to nitrocellulose (Bio-Rad catalog number 162-0112; Invitrogen (catalog number LC2000). The membranes were blocked in SuperBlock blocking buffer (Pierce, catalog number 37515) for 1 h and incubated with appropriate primary and secondary antibodies. If the primary or secondary antibody was HRP-conjugated, ECL Plus Western blotting detection reagents (GE Healthcare Biosciences, catalog number RPN2132) were used and developed on Kodak BioMax light film (Z370371). If the secondary antibodies were IRDye 800 or IRDye 680, the Odyssey infrared (LI-COR Biosciences) system was used to analyze the results.

##### siRNA Experiments

siGENOME SMARTpool Human NEDD4 (catalog number M-007178-02-0005), siGENOME siRNA, human NEDD4 (catalog number D-007178-03-0005 and catalog number D-007178-04-0005), and siGENOME nontargeting siRNA Pool #1 (catalog number D-001206-13-05) were purchased from Thermo Scientific (Dharmacon). Lipofectamine RNAiMAX (Invitrogen catalog number 13778) was diluted in OPTI-MEM (Invitrogen catalog number 31985-070) prior to transfection of the cells. The cells were lysed 48–72 h after transfection.

## RESULTS

### 

#### 

##### PRRG4 Localizes to the Plasma Membrane

Although membrane localization of PRRG4 is predicted by sequence properties, this has not previously been investigated. We used V5 antibodies to examine the localization of the transfected PRRG4-V5-His (supplemental Fig. 1*A*) in human embryonic kidney 293 cells and in T47D breast carcinoma cell (supplemental Fig. 2*A*). PRRG4 membrane localization was further investigated by cell surface biotinylation and purification with NeutrAvidin agarose resin. Overexpressed PRRG4 was detected in the plasma membrane-enriched fraction but not in the cytosolic-enriched fraction (supplemental Fig. 2*B*). These results are consistent with a previous study reporting that the related protein PRRG2 is localized to the plasma membrane (16).

##### Possible Cytosolic Binding Partners of PRRG4

PRRG4 contains potential WW and SH3 binding motifs (2). Therefore, we sought to identify WW and SH3 domains that interact with PRRG4. The cytosolic portion of PRRG4 (PRRG4-cyto) with V5, His, and HA tags (supplemental Fig. 1*B*) was incubated with WW or SH3 arrays containing WW or SH3 domains from different proteins immobilized on membranes. Each WW or SH3 domain is immobilized on the membrane in duplicate. 67 different WW domains and 130 SH3 domains from human proteins were tested for PRRG4 binding. Interacting proteins are observed as dark spots by chemiluminescent detection (supplemental Fig. 3, *A* and *B*).

10 proteins from the WW array and 15 proteins from the SH3 array were found to interact with cytosolic PRRG4 (Table 1, supplemental Table 1, supplemental Fig. 3, *A* and *B*). The proteins identified from the WW arrays include two proteins that were shown to interact with PRRG2 in previous studies (15, 16), YAP and NEDD4. However, several additional possible binding partners were identified for the first time. These proteins are members of the membrane-associated guanylate kinase (MAGUK) family, MAGI-1 (BAIAP1), MAGI-2 (AIP-1), MAGI-3; another member of the Hippo pathway, WWTR1 (TAZ); and several E3 ubiquitin-protein ligases including, NEDD4L, ITCH, WWP1, and NEDL1. Most of the hits for the SH3 array are nonreceptor tyrosine kinases or proteins that regulate cytoskeletal proteins (Table 1 and supplemental Table 1).

**TABLE 1:** **Summary of protein-protein interactions** This table summarizes all interacting partners that were further tested in this study. Additional proteins identified on domain array screen but not further tested are listed in supplemental Table I. Co-IP, co-immunoprecipitation.

Symbol	Name	Array	Binds to PRRG4 in GST pulldown	Binds to PRRG4 in Co-IP	Binds to PRRG2
MAGI-1	Membrane-associated guanylate kinase-related	WW	YES	YES	YES
MAGI-3	Membrane-associated guanylate kinase-related	WW	YES	Not tested*^a^*	Not tested
YAP	Yes-associated protein	WW	YES	YES	YES*^b^*
TAZ/WWTR1	Transcriptional co-activator with PDZ binding motif	WW	YES	YES	YES
NEDD4L	E3 ubiquitin-protein ligase	WW	YES	Not tested*^a^*	YES
NEDD4	E3 ubiquitin-protein ligase	WW	YES	Not tested*^a^*	YES*^c^*
Lyn	Src family of non-receptor protein tyrosine kinase Lyn	SH3	NO	Not tested*^a^*	Not tested
OSTF	OSF: osteoclast-stimulating factor 1	SH3	NO	NO	Not tested
Grb2	Growth factor receptor-bound protein 2	SH3	Not tested*^d^*	NO	Not tested
βpix/cool1/Y124	PAK-interacting exchange factor β	SH3	NO	NO	Not tested

*^a^* MAGI-3, NEDD4L, and Lyn constructs are not FLAG-tagged.

*^b^* Source: this study and a previous study (16).

*^c^* Source: previous study (15).

*^d^* The size of Grb2 is close to GST. Western blot gives non-specific strong bands.

Because the arrays only include the WW or SH3 domain and not the entire possible interacting proteins, we sought to confirm their interaction with PRRG4 at the whole protein level. We selected YAP1, YAP2, WWTR1, MAGI-1, MAGI-3, NEDD4, and NEDD4L from the WW array and OSTF, cool1/βPIX, Grb2, and Lyn from the SH3 array. These selections were based on strength of signal, availability of reagents to enable further study, and desire to study proteins that represent different classes of binding partners. The other hits from the screen that were not studied further are listed in supplemental Table 1.

**FIGURE 1.**
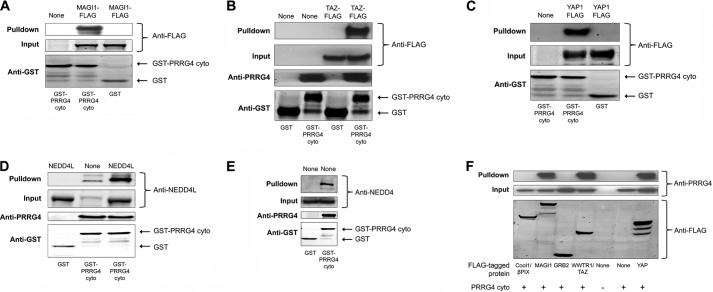
**GST pulldown and co-immunoprecipitation experiments confirm findings from the arrays.** *A–E*, GST pulldown was done to confirm the interaction of PRRG4-cytosolic domain with: MAGI-1 (*A*); WWTR1/TAZ (*B*); YAP (*C*); NEDD4L (*D*); and NEDD4 (*E*). WWTR1/TAZ, MAGI-1, and YAP are FLAG-tagged, and anti-FLAG antibody was used for Western blot. Overexpressed proteins are written at the top of lanes. The cytosolic domain of PRRG4 interacts with endogenous NEDD4L (*D*) and NEDD4 (*E*) in GST pulldown experiments. *F*, co-immunoprecipitation experiments show that the cytosolic domain of PRRG4 interacts with WW domain-containing YAP, WWTR1, and MAGI-1b but not with SH3 domain-containing Grb2 or cool1/βPIX. Anti-FLAG antibody was used to immunoprecipitate the FLAG-tagged proteins, and anti-PRRG4 antibody was used for immunoblotting the pulled down PRRG4. Co-expressed cytosolic PRRG4 could be co-immunoprecipitated with MAGI, YAP, and TAZ from HEK293 cell lysates but not with Grb2 or cool1/βPIX.

We made GST-tagged cytosolic PRRG4 (GST-PRRG4cyto, supplemental Fig. 1*C*) and performed GST pulldowns. We blotted with the antibodies against the interacting proteins to confirm the pulldown. All the tested WW domain-containing proteins (YAP1, YAP2, WWTR1, MAGI-1, MAGI-3, NEDD4, NEDD4L) bound to GST-PRRG4cyto but not to the GST-only control (Fig. 1, *A–E*). However, none of the possible binding partners identified from the SH3 array that we tested could be pulled down via GST-PRRG4cyto in the same experimental conditions. It is worth noting that we could pull down endogenously expressed NEDD4 and NEDD4L with GST-PRRG4cyto as well (Fig. 1, *D* and *E*, and supplemental Fig. 4). To show the specificity of the signal from the endogenous NEDD4, we used an siRNA strategy to knock down NEDD4 proteins in 293 cells. Endogenous NEDD4 binding to GST-PRRG4cyto decreased significantly upon siRNA treatment, showing the specificity of the antibody (supplemental Fig. 4). We could also detect that binding of endogenous NEDD4L proteins to the cytosolic PRRG4 and the bound NEDD4L proteins increase upon overexpression of the NEDD4L protein (Fig. 1*D*).

We next tested some of these interactions by co-immunoprecipitation. Cytosolic PRRG4 in a mammalian expression vector (supplemental Fig. 1*D*) was co-expressed with FLAG-tagged proteins (YAP1, YAP2, WWTR1, MAGI-1b, OSTF, cool1/βPIX, Grb2). Similar to GST pulldown results, WW-containing candidate proteins could be co-immunoprecipitated in this assay but not the SH3-containing ones (Fig. 1*F*).

##### Sequence Analysis Reveals a Second WW Binding Motif

**FIGURE 2.**
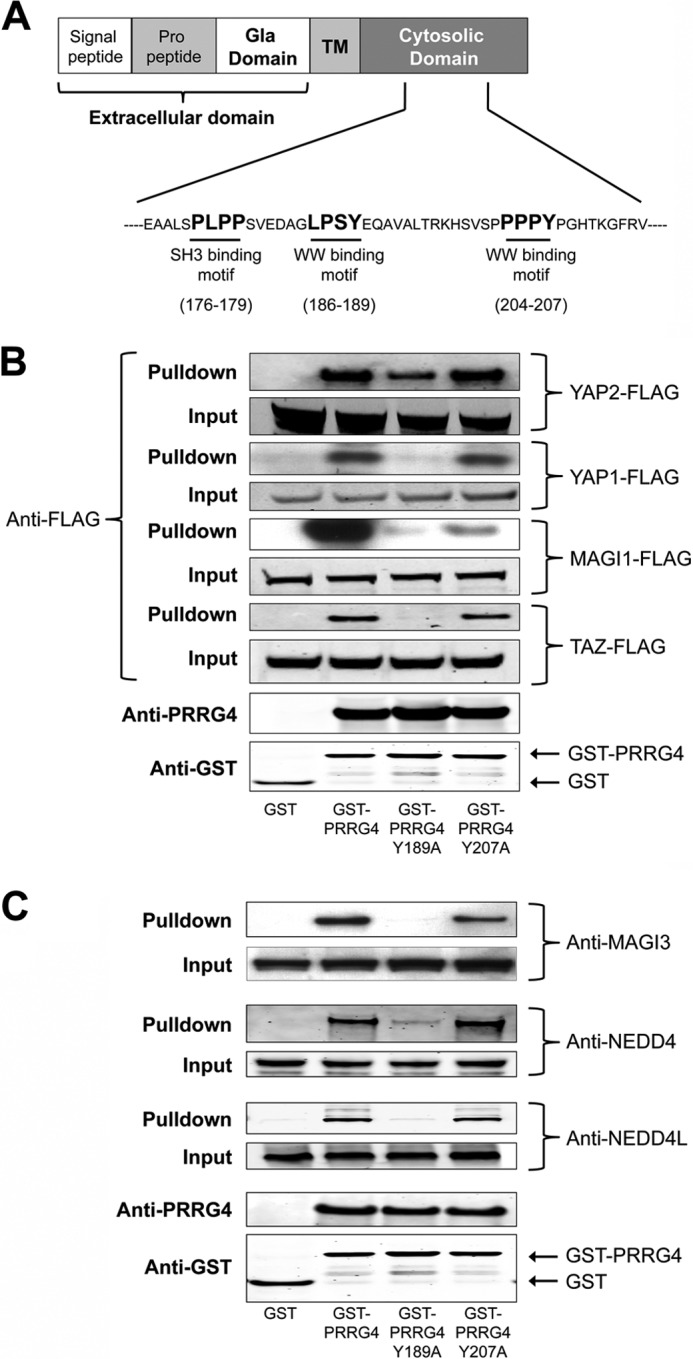
**Novel WW interaction motif on PRRG4.** *A*, sites of novel WW interaction motif (186–189) and previously predicted WW (204–207) and SH3 (176–179) interaction motifs (2). *TM*, transmembrane. *B*, pulldown results using wild type and mutated cytosolic domains. *First lane*, GST only; *second lane*, GST-PRRG4 = wild type construct; *third lane*, GST-PRRG4 Y189A = cytosolic domain in which putative WW domain is mutated to LPSA; *fourth lane*, GST-PRRG4 Y207A = cytosolic domain construct in which putative WW domain is mutated to PPPA. Mutation of (L/P)P*X*Y motif at 186–189 results in loss or reduction of binding of PRRG4 cytosolic domain with TAZ, YAP1, YAP2, and MAGI-1 proteins. Mutation of PP*X*Y motif at 204–207 results in reduction of binding to MAGI-1 only. *C*, mutation of (L/P)P*X*Y motif at 186–189 results in loss or reduction of binding of PRRG4 cytosolic domain with MAGI-3, NEDD4, and NEDD4L proteins. Mutation of PP*X*Y motif at 204–207 does not affect binding to these partners.

A PP*X*Y WW domain binding motif is present in the PRRG4 sequence at amino acids 204–207. This is the WW domain binding motif that was suggested in the original study (2). However, leucine can substitute for the first proline, and LP*X*Y is also considered a WW binding motif. Sequence analysis reveals the presence of an LP*X*Y motif at amino acids 186–189 (Fig. 2*A*). Mutating the tyrosine of (L/P)P*X*Y motif to alanine inactivates the WW binding motif. The tyrosine residue in both of these WW domain binding motifs was mutated to alanine, thus forming PRRG4-Y189A or PRRG4-Y207A. The mutants were then screened for binding to the identified candidates to determine which motif in PRRG4 facilitates the interaction.

Our results show that the newly identified WW binding motif LPSY (186–189) is the main binding site for YAP, TAZ, NEDD4, and NEDD4L (Fig. 2, *B* and *C*). Although the LPSY (186–189) motif seems to be more crucial, PPPY (204–207) motifs are also important for PRRG4 interaction with MAGI-1 and MAGI-3 interaction (Fig. 2, *B* and *C*).

**FIGURE 3.**
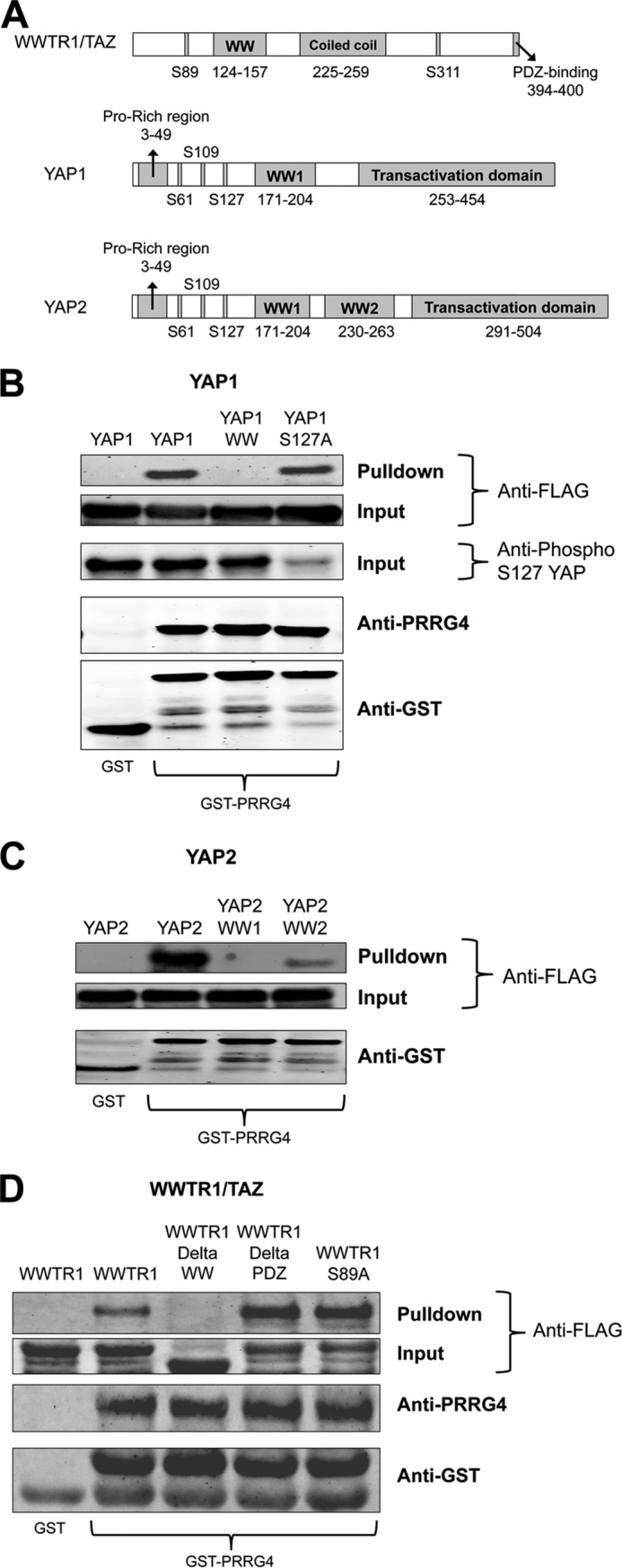
**Effects of mutations of WW domains of YAP and WWTR1/TAZ on binding to PRRG4.** *A*, domains of WWTR1/TAZ, YAP1, and YAP2 are shown. YAP2 has an additional WW domain between residues 230 and 263. *B*, *GST*, GST only; *GST-PRRG4*, cytosolic domain of PRRG4-tagged with GST; *YAP1*, wild type YAP1; *YAP1 WW*, WW domain of YAP1 is mutated by converting tryptophan 199 to alanine and proline 202 to alanine; *YAP1 S127A*, serine 127 of YAP1 is mutated to alanine. Mutation of YAP1 WW domain results in reduction in binding to PRRG4. Mutation of S127A has no impact on binding. Anti-phospho-Ser-127 (*Anti-phospho S127*) antibodies weakly recognize the YAP1 S127A as expected. *C*, YAP2 has two WW domains, both required for efficient binding to PRRG4. *YAP2*, wild type YAP2 protein; *YAP2 WW1*, first WW domain is mutated (tryptophan 199 to alanine and proline 202 to alanine); *YAP2 WW2*, second WW domain is mutated (tryptophan 258 to alanine and proline 261 to alanine). Mutation of either WW domain of YAP2 results in decreased GST-PRRG4 binding. *D*, *WWTR1*, wild type WWTR1/TAZ protein; *WWTR1DeltaWW*, WW domain (amino acids 111–158) is deleted; *WWTR1DeltaPDZ*, PDZ domain (amino acids after 393) is deleted; *WWTR1-S89A*, serine 89 is mutated to alanine. Binding of WWTR1/TAZ to GST-PRRG4 is decreased in the WW domain deleted form but not in the S89A nor the DeltaPDZ forms.

We further examined the interaction between YAP and PRRG4. YAP1 has only one WW domain (Fig. 3*A*), and mutation of this domain reduces PRRG4-YAP1 binding (Fig. 3*B*). On the other hand, YAP2 has two WW domains (Fig. 3*A*), and both of the domains are important for interaction (Fig. 3*C*). YAP interacts with 14-3-3 upon phosphorylation of the serine residue at 127. When we used YAP1 with a serine 127 to alanine mutation, the YAP-PRRG4 interaction occurred comparable with wild type (Fig. 3*B*), showing that phosphorylation of this residue is not important for PRRG4-YAP interaction.

Similar to YAP-PRRG4 interaction, deletion of the WW domain of WWTR1/TAZ diminishes binding to PRRG4 (Fig. 3*D*). However, neither the deletion of the PDZ binding domain nor the mutation of serine 89 to alanine (Fig. 3*D*) had any clear effect on PRRG4-WWTR1/TAZ interactions.

##### Expression Profiles of PRRG4 and PRRG2

**FIGURE 4.**
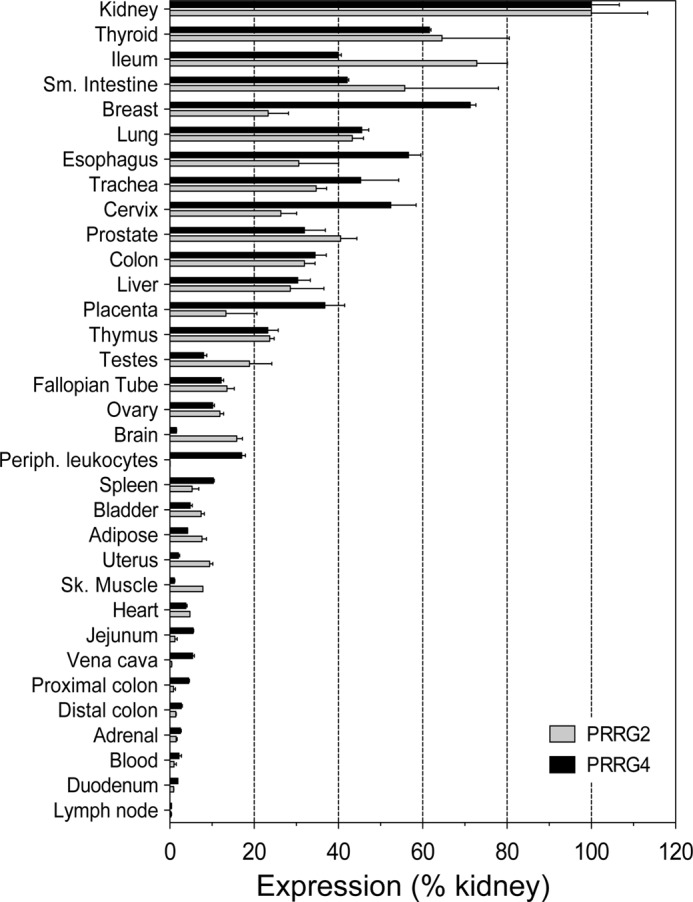
**Expression of PRRG4 and PRRG2 in multiple tissues.** PRRG4 and PRRG2 expression in 33 human tissues was tested. *Sm. Intestine*, small intestine; *Periph. leukocytes*, peripheral leukocytes; *Sk. Muscle*, skeletal muscle.

Both PRRG2 and PRRG4 are expressed in a variety of tissues according to the original studies (1, 2). However, quantitative expression in different tissues has not been studied. We determined relative PRRG4 or PPRG2 RNA expression in 33 human tissues (Fig. 4) by quantitative RT-PCR. As was found in the original studies (1, 2), both are expressed ubiquitously among the tissues tested. The highest expression was observed in kidney; expression in other tissues is shown normalized to expression levels in kidney. There is up to 200-fold variation in expression levels among the tissues examined, ranging from the highest (kidney, thyroid, breast, lung, etc.) to the lowest (uterus, jejunum, proximal colon, duodenum, etc.). In general, relative expression levels of PRRG4 and PRRG2 are concordant in most of the tissues, meaning that both are expressed at high, moderate, or low levels. There are some exceptions to this; breast has high PRRG4 expression and moderate PRRG2 expression, and brain has moderate PRRG2 expression and low PRRG4 expression.

##### Vitamin K Effect on PRRG4 Protein

The Gla protein domain is characterized by glutamate residues that are post-translationally modified by vitamin K-dependent carboxylation to form γ-carboxyglutamate. Monoclonal antibodies against Gla recognize only the γ-carboxylated form but not the uncarboxylated form of the Gla domain. We tested the post-translational modification of the Gla domain of PRRG4 in medium with vitamin K or warfarin, an inhibitor of vitamin K cycling. Gla antibody can immunoprecipitate full-length PRRG4 protein upon vitamin K treatment but not warfarin treatment, showing that PRRG4 undergoes vitamin K-dependent post-translational modification as expected (supplemental Fig. 5*A*). Furthermore, a slower migrating form of PRRG4 accumulates upon warfarin treatment, which may reflect loss of the γ-carboxylated residues (supplemental Fig. 5*B*).

## DISCUSSION

Although it has been more than 15 years since the initial identification of PRRG family members, the function of these Gla domain-containing membrane proteins is still unknown. As one strategy to probe the function of these proteins, we analyzed their protein-protein interactions and present here the most comprehensive such analysis to date. PRRG4, which is similar to PRRG2 by sequence analysis, has putative SH3 and WW binding motifs in the cytosolic domain. In the work shown here, we identified several binding partners of PRRG4. Several of the interacting proteins identified via WW array screen were confirmed by GST pulldown and co-immunoprecipitation experiments. However, we could not confirm any of the PRRG4 interaction candidates from the SH3 array screen with the same methods. There are several other WW and SH3 proteins that were identified in the screen but have not been studied further here (supplemental Table 1). Before ruling out the SH3-interacting motif of PRRG4 as nonfunctional, it may be useful to do further binding experiments involving these other candidate proteins identified in the screen.

Phosphorylation of certain serine residues is important for the binding of Hippo pathway proteins to 14-3-3 and for proper localization in the cell. S127A mutation of YAP and S89A of WWTR1/TAZ did not affect PRRG4 binding (Fig. 3, *B* and *D*), meaning that this phosphorylation is not crucial for WW binding.

The role of PRRG proteins in the nervous system also needs to be studied further. A study of WAGR syndrome revealed that PRRG4 is one of only three genes deleted in all of the patients tested (12). Whether PRRG4 has any role in mental retardation and autism might be identified by investigating further the function of PRRG4 protein in the nervous system. Among the PRRG4- and PRRG2-interacting proteins that we found in this study, MAGI proteins are particularly interesting in this regard. MAGI-1, -2, and -3 all contain WW domains and were confirmed as PRRG4-binding proteins in our experiments. MAGI proteins are members of the membrane-associated guanylate kinase family with inverted domain organization, and they participate in the assembly of multiprotein complexes at cell junctions (22). Studies in *Caenorhabditis elegans* showed that MAGI-1 proteins are important for memory consolidation and associative learning (23). The finding here might explain the learning defect of WAGR patients, all of whom carry a deletion of one allele of the PRRG4 gene (12). The PRRG4-MAGI interaction should be further studied in terms of the effects of PRRG proteins on synaptic functions of the neurons. Although we know that PRRG2 is also expressed in the brain (Fig. 4), more precise localization of gene expression within the brain may also shed light on functions of the protein.

A recent study on mesenteric lymph node gene expression profiles of BALB/c mice in response to three common food allergens identified PRRG4 as one of the top 10 up-regulated genes (13). The three allergens used in this study were β-lactoglobulin (BLG) from cow's milk, ovalbumin (OVA) from hen's egg white, and peanut agglutinin (PNA). Whether any of the PRRG4-interacting proteins that we identified here is essential for this observed allergen response is of interest for further studies.

Sequence analysis shows that PRRG2 and PRRG4 closely resemble each other, whereas PRRG1 and PRRG3 are similar to each other (2). Although we showed that the PRRG4-interacting partners can also bind to PRRG2 (supplemental Fig. 6), it will also be of interest to determine whether PRRG1 and PRRG3 interact with these proteins.

The function of the Gla domain is not covered in this study. We have found that PRRG4 proteins from cells grown in vitamin K-containing medium can be recognized by anti-Gla antibodies at immunoblotting experiments (supplemental Fig. 5*A*). Furthermore, immunoblots with anti-Gla and anti-PRRG4 antibodies resulted in multiple bands depending on the vitamin K levels in the medium (supplemental Fig. 5*B* and data not shown). These multiple bands are likely due to the post-translational modification of the Gla domain. However, whether these modifications have any effect on cytoplasmic signaling is not known. The possible effect of Gla maturation on the WW protein interactions and the extracellular ligands for PRRG proteins should be studied further.

This study is the most comprehensive to date of PRRG family signal transduction. Further analysis of the PRRG4-interacting proteins identified in this study may help to elucidate the role of PRRG4 in cancer, allergy, and neurological disorders.

## Footnotes

2 The abbreviations used are:

PRRG: proline-rich γ-carboxyglutamic acid
Gla: γ-carboxyglutamic acid
WAGR: Wilms tumor, aniridia, genitourinary malformations and mental retardation
SH: Src homology
MAGI: membrane-associated guanylate kinase, WW and PDZ domain-containing protein 1
OSTF: osteoclast-stimulating factor 1
YAP: Yes-associated protein
Bis-Tris: 2-(bis(2-hydroxyethyl)amino)-2-(hydroxymethyl)propane-1,3-diol
cyto: cytosolic domain
TAZ: transcriptional co-activator with PDZ binding motif.
